# Phylogenetic Analysis of Highly Pathogenic Avian Influenza H7 Viruses in Australia and New Zealand Suggests Local Viral Evolution

**DOI:** 10.3390/vetsci12121208

**Published:** 2025-12-17

**Authors:** Jesiaman Silaban, Stephen Ogada, Muhammad Noman Naseem, Yun Hee Baek, Min-Suk Song, Sheila Cecily Ommeh

**Affiliations:** 1 School of Chemistry and Molecular Biosciences, The University of Queensland, Brisbane, QLD 4072, Australia; 2 Queensland Alliance for Agriculture & Food Innovation, Centre for Animal Science, The University of Queensland, Brisbane, QLD 4072, Australia; 3 School of Veterinary Science, The University of Queensland, Gatton, QLD 4343, Australia; 4 Department of Microbiology, College of Medicine and Medical Research Institute, Chungbuk National University, Cheongju 28644, Chungbuk, Republic of Korea; microuni@chungbuk.ac.kr (Y.H.B.);

**Keywords:** genomic surveillance, HPAI, infectious disease, One-Health, pandemic, zoonosis

## Abstract

Avian Influenza (AI) remains a critical threat to livestock production in Oceania, with recent outbreaks of Highly Pathogenic Avian Influenza (HPAI) H7 subtypes in Australia and New Zealand. Historically, AI detections in these countries were largely confined to wild birds. However, since 2024, multiple outbreaks in commercial poultry populations have been reported, with the emergence of HPAI H7N3, H7N8, and H7N9 in Australia and H7N6 in New Zealand. To investigate the epidemiological links of these outbreaks, we retrieved all publicly available H7 hemagglutinin (HA) gene sequences from animals in Oceania between 2014 and 2025 that were deposited in GISAID and NCBI databases. Phylogenetic reconstruction using maximum likelihood methods revealed distinct Australian and New Zealand clades, suggesting local viral evolution rather than recent transboundary introductions. The absence of phylogenetic clustering between Oceanian and Asian H7 sequences suggests limited recent gene flow between regions, particularly in the HA gene segment. These local viral mutations can pose a threat to the poultry industry; therefore, continuous genomic surveillance in commercial farms and wild bird reservoirs is crucial. Such efforts support the One Health framework by enabling early detection of pathogenic variants and reducing zoonotic spillover risks for animal and public health.

## Simple Summary

Avian Influenza, a disease caused by influenza A viruses, primarily affects birds. However, this disease can sometimes spread to humans, particularly through contact with infected livestock, such as poultry, and poses a serious threat to both animal and human health, as well as food security. Highly Pathogenic Avian Influenza subtypes (HPAI), like H5 and H7, spread fast and can cause severe disease with high mortality. Recently, outbreaks of HPAI H7 subtypes have occurred in Australia and New Zealand, posing a significant challenge to livestock production in these countries. While this virus was largely confined to wild birds, multiple outbreaks have been reported in commercial farms since 2024. In this study, we investigated HPAI H7 subtype viruses associated with recent outbreaks in livestock across Oceania, with a particular focus on Australia and New Zealand, to identify their possible links. After genetic relatedness analysis, HPAI H7 subtype viruses from Australia and New Zealand were found to be distinct from those in neighbouring regions of East and Southeast Asia, suggesting they were local. Although more farm outbreaks were reported earlier this year, successful eradication was achieved by mid-year, highlighting the importance of ongoing surveillance, coordinated national response, and improved biosecurity measures.

## 1. Introduction

Avian Influenza (AI) is a highly contagious viral disease that primarily affects birds, with wild aquatic species serving as the natural reservoir of the virus. Avian influenza is not restricted to avian hosts, as mammals, including humans, can also become infected, classifying AI as a zoonotic disease [1,2]. The disease poses a significant threat to animal health and food security, especially when caused by Highly Pathogenic Avian Influenza (HPAI) strains. HPAI spreads rapidly within flocks, leading to high morbidity and widespread mortality, particularly in commercial poultry systems [3]. The transmission of AI occurs through direct and indirect contact with infected animals. The most effective transmission routes are direct contact and airborne spread [4,5]. However, indirect transmission via contaminated equipment, clothing, vehicles, or during transportation can contribute significantly to the spread of the disease [6]. Clinical signs in infected birds vary widely, depending on the virulence of the strain and the host species. Common symptoms include respiratory distress, gastrointestinal disorders, and neurological signs. In commercial poultry, such as laying hens, reduced egg production is a notable indicator of infection [7,8].

AI is caused by the Avian Influenza Virus (AIV), an enveloped, negative-sense single-stranded RNA virus belonging to the *Alphainfluenzavirus* genus within the *Orthomyxoviridae* family [9]. Like other influenza viruses, AIV has a segmented genome with eight RNA segments. These segments encode two surface glycoproteins: hemagglutinin (HA) and neuraminidase (NA), and six internal proteins: polymerase subunits (PB2, PB1, PA), nucleoprotein (NP), matrix protein (MP), and non-structural proteins (NS) [10,11].

The genetic diversity of AIV is primarily defined by the HA and NA segments. HA mediates viral attachment to host cells, while NA facilitates viral release and spread [12]. To date, 18 HA (H1–H18) and 11 NA (N1–N11) subtypes have been identified, resulting in various combinations, such as H5N1 and H7N9, that form distinct AIV subtypes [13]. These two genes are central to subtype classification, with the HA gene playing a key role in determining viral lineage and clade. Pathogenicity has also been used to classify AIVs, which is then assessed through in vivo testing and molecular characterisation of the HA protein [14]. This classification distinguishes between Low Pathogenic Avian Influenza (LPAI) and Highly Pathogenic Avian Influenza (HPAI) [15]. HPAI strains, usually of the H5 and H7 subtypes, cause severe disease, spread rapidly, and result in high mortality. In contrast, LPAI viruses, which can belong to any HA subtype (H1–H18), generally cause mild or asymptomatic infections.

Oceania, a geopolitical region comprising Australia, New Zealand, Fiji, Papua New Guinea, and other Pacific islands, presents a unique epidemiological setting for AIV due to its distinct geographic, ecological, and poultry industry characteristics [16]. While AI has been detected in livestock across the region, comprehensive surveillance data remain limited outside of Australia and New Zealand. Australia has a history of both LPAI and HPAI outbreaks in poultry [17]. Detected LPAI subtypes include H5 and H7 LPAI, as well as non-H5 or non-H7 subtypes, such as H9N2, H4N8, H6N4, and H10N7 [18]. Although no HPAI H5 outbreaks have been reported in Australian livestock [19], multiple HPAI H7 outbreaks have occurred. Historical HPAI events were caused by subtypes such as H7N7, H7N3, H7N4, and H7N2. More recently, since 2024, Australia has experienced additional HPAI H7 outbreaks across several states, with H7N3, H7N8, and H7N9 identified as the causative subtypes. New Zealand, in contrast, confirmed its first case of HPAI in poultry in 2024, involving the H7N6 subtype [20]. Previously, only LPAI viruses had been detected, predominantly in wild birds, where infections were generally asymptomatic [21]. A notable commonality in recent outbreaks in both countries is the involvement of HPAI H7 subtypes, highlighting a common subtype-driven epidemiological pattern. However, gaps remain in understanding their epidemiological and transmission dynamics. Current studies provide limited insight into the phylogenetic relationships among recent outbreaks, and no comprehensive re-analysis has been conducted to clarify epidemiological links.

This study aimed to provide an update on the status of HPAI H7 subtype viruses responsible for recent outbreaks in livestock across Oceania, with a particular focus on Australia and New Zealand. The main objective was to assess the recent HPAI H7 epidemiological links, and to discuss the existence of their threat to livestock production.

## 2. Materials and Methods

### 2.1. Avian Influenza Virus Sequence Retrieval

Ff Complete hemagglutinin (HA) gene sequences of Avian Influenza Virus (AIV) subtype H7 were retrieved from the Global Initiative on Sharing All Influenza Data (GISAID) database (https://platform.epicov.org/) (accessed on 10 June 2025). Sequence selection criteria included influenza A viruses isolated from animal hosts within Oceania, with collection dates between 1 January 2014, and 31 March 2025, to represent data from the past decade (Supplementary Table S1). To provide regional context, additional H7 subtype sequences from Southeast Asia (Brunei, Cambodia, Indonesia, Laos, Malaysia, Myanmar, Philippines, Singapore, Thailand, Vietnam) and East Asia (China, South Korea, North Korea, Japan, Mongolia, Macau, Taiwan) were obtained by filtering location metadata and manual curation (Supplementary Tables S2 and S3). Partial sequences and incomplete HA gene entries were excluded from the analysis. Associated metadata, including virus nomenclature, subtype, collection date, geographic origin, and host species, were also collated. To ensure comprehensive data acquisition, a parallel search was conducted in the NCBI Influenza Virus Database (https://www.ncbi.nlm.nih.gov/genomes/FLU/Database/nph-select.cgi) (accessed on 10 June 2025), focusing on H7 subtype AIV isolates from animal hosts in Oceania within the same temporal range.

### 2.2. Phylogenetic Analysis

HA sequences were aligned using MAFFT version 7.490 (Osaka University, Suita, Japan) [22] with 1000 iterative refinements to optimise alignment quality. The resulting multiple sequence alignment (MSA) was visually inspected and manually curated using AliView version 1.28 (Uppsala University, Uppsala, Sweden) [23]. Sequences exhibiting excessive length variability, ambiguous nucleotides, or significant gaps were trimmed or excluded to improve the reliability of downstream phylogenetic analyses. Phylogenetic relationships were inferred via maximum likelihood (ML) methods implemented in IQ-TREE version 2.1.4 (University of Vienna, Vienna, Austria) [24]. The best-fit nucleotide substitution model was identified automatically using IQ-TREE’s Model Finder algorithm. ML tree reconstruction was performed using 100,000 bootstrap replicates to assess the robustness of node support. Phylogenetic trees were visualised and refined using FigTree version 1.4.4 (Institute of Evolutionary Biology, University of Edinburgh, Edinburgh, UK) [25], with adjustments to taxa labels, branch aesthetics, and the inclusion of informative legends to enhance interpretability. Time-scaled phylogenetic tree was constructed using BEAST version 1.10.4 and maximum credibility lineage trees were generated using TreeAnnotator version 1.8 included in the BEAST package (University of Auckland, Auckland, New Zealand) [26]. To validate sequence identities and support phylogenetic inferences, the Basic Local Alignment Search Tool (BLAST) version 2.17.0 (National Center for Biotechnology Information, Bethesda, MD, USA) from NCBI (https://blast.ncbi.nlm.nih.gov) was employed (accessed on 14 July 2025). BLAST analyses facilitated the identification of closely related sequences and aided in the contextual interpretation of phylogenetic clustering patterns.

## 3. Results

### 3.1. Summary of Avian Influenza H7 Sequence from Oceania

The availability of Avian Influenza Virus (AIV) H7 sequences from Oceania was limited, with all publicly accessible sequences originating solely from Australia and New Zealand. No other countries in the region had submitted AIV H7 subtype sequences to the GISAID or NCBI Influenza Virus databases. A total of 15 complete HA gene sequences from Oceania were retrieved (Table 1). New Zealand contributed four isolates: two H7N7 viruses from mallard ducks in 2015 and two H7N6 viruses from chickens in 2024. Australia contributed eleven isolates, exhibiting greater subtype diversity derived from both wild waterfowl and poultry across multiple states.

In addition, 987 AIV H7 sequences were retrieved from Southeast and East Asia (Table 2). Southeast Asia contributed 12 sequences exclusively from Cambodia (*n* = 10) and Vietnam (*n* = 2), while East Asia contributed 975 sequences, predominantly from China (*n* = 865), with additional sequences from Hong Kong, Japan, North Korea, South Korea, Mongolia, and Taiwan.

### 3.2. Phylogenetic Analysis of AIV H7 in Australia and New Zealand

**Figure 1:**
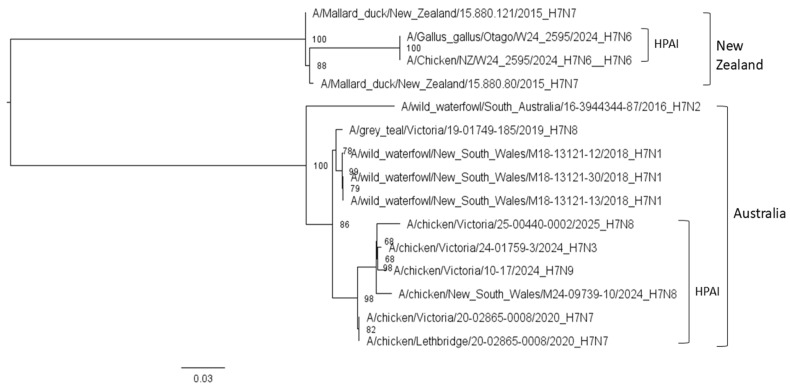
Maximum likelihood phylogenetic tree of AIV H7 HA gene sequences from Australia and New Zealand. The scale bar indicates nucleotide substitutions per site. HPAI: Highly Pathogenic Avian Influenza.

Phylogenetic reconstruction using maximum likelihood (ML) methods was performed on aligned HA gene sequences from Australia and New Zealand. The resulting phylogeny revealed two well-supported, geographically distinct clades corresponding to Australian and New Zealand isolates, indicating no recent common ancestry within the sampled timeframe (Figure 1). Notably, HPAI H7 viruses from 2024 and 2025 clustered into separate subgroups within their respective regional clades.

**Figure 2:**
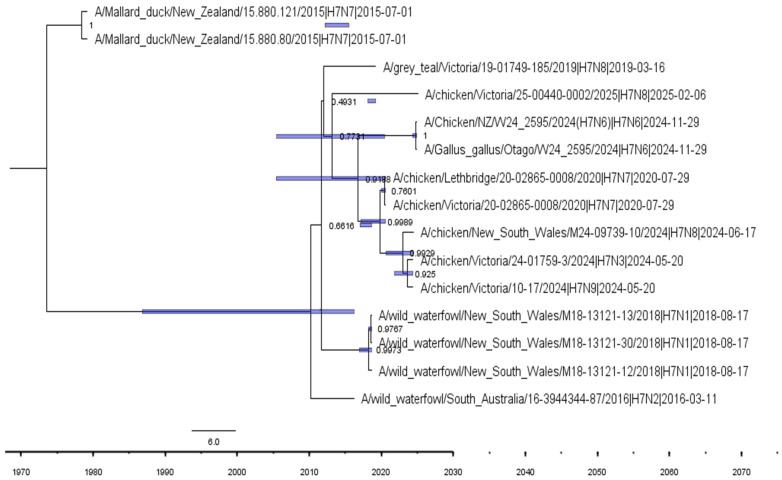
Time-scaled phylogenetic tree of AIV H7 HA gene sequences from Australia and New Zealand. The blue horizontal bars represent 95% Highest Posterior Density (HPD) intervals.

Time-scaled phylogenetic analysis revealed that the 2024–2025 Australian and New Zealand H7 poultry outbreaks originated from a common ancestor circulating in wild birds around 2019 (Figure 2). The presence of multiple NA subtypes within a single H7 backbone indicates extensive reassortment following spillover into poultry. Earlier New Zealand (2015) and New South Wales (2018) H7 lineages formed distinct, extinct clades, demonstrating multiple independent incursions rather than sustained local persistence.

**Figure 3:**
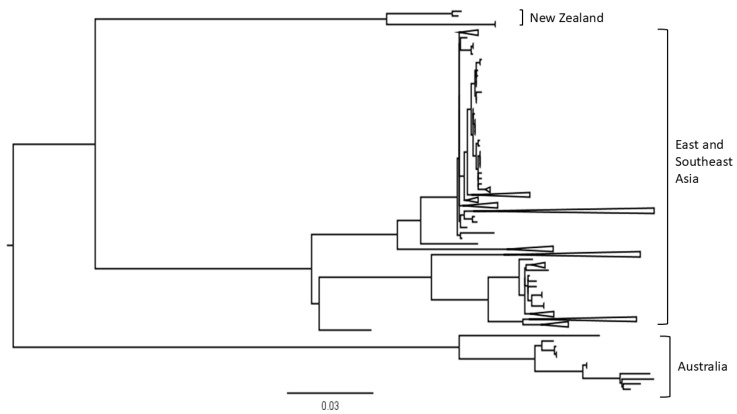
Maximum likelihood phylogenetic tree of AIV H7 HA gene sequences from Oceania, Southeast Asia, and East Asia.

To contextualise the genetic diversity of HA gene sequences from Oceania within a broader regional framework, HA sequences from Southeast and East Asia were incorporated into the Oceania dataset, and a maximum likelihood phylogenetic tree was generated. The resulting tree also demonstrated that AIV H7 viruses from Australia and New Zealand formed distinct, well-separated clades, while East Asian sequences exhibited substantial genetic diversity. Southeast Asian sequences were interspersed within East Asian clades, indicating regional connectivity (Figure 3).

Along with analysing the haemagglutinin (HA) gene, the neuraminidase (NA) genes from highly pathogenic avian influenza (HPAI) H7Nx viruses linked to recent outbreaks in Australia and New Zealand were also examined. This analysis aimed to find the most closely related neuraminidase sequences of avian influenza viruses available in the NCBI GenBank database using the BLAST algorithm. A total of six HPAI H7Nx virus sequences from outbreaks reported in Australia and New Zealand between 2024 and 2025 were retrieved (Table 3). These isolates corresponded to four distinct subtypes: H7N6, H7N9, H7N3, and H7N8.

To determine the similarity of the NA segments, BLAST analyses were conducted for each neuraminidase gene (N6, N9, N3, and N8). BLAST results for the N6 sequences of the two H7N6 isolates from New Zealand (A/Chicken/NZ/W24_2595/2024 and A/Gallus gallus/Otago/W24_2595/2024) indicated high similarity (>90% identity) with LPAI H4N6 viruses isolated from wild birds in New Zealand in 2004 and 2005. The NA gene from the H7N9 isolate (A/chicken/Victoria/10-17/2024) shared more than 95% identity with wild bird-derived sequences from Australia collected between 2017 and 2023. These included H5N9 and H11N9 subtypes. The N3 gene from the H7N3 isolate (A/chicken/Victoria/24-01759-3/2024) showed high identity (>97%) with LPAI viruses isolated from wild birds in Australia. These included subtypes H5N3, H10N3, and H1N3. The N8 gene from the H7N8 isolates (A/chicken/New South Wales/M24-09739-10/2024 and A/chicken/Victoria/25-00440-0002/2025) showed top BLAST hits with AIVs from East Asia, including South Korea, Japan, and China, ranging from 97.30% to 98.09% of identities. In addition, one of the top matches with 97.38% identity was detected with an isolate from Thailand, located in Southeast Asia. All matches were with non-H7 subtypes such as H3N8, H6N8, and H4N8.

## 4. Discussion

Avian Influenza Virus (AIV) has been circulating among avian populations in Oceania for decades, particularly in Australia and New Zealand. Historically, AIV detection in both countries has predominantly occurred in wild bird populations, with limited evidence of significant transmission in domestic poultry [19,21]. However, in 2024, a notable shift occurred with multiple outbreaks of HPAI H7 reported in commercial poultry in Australia [18] and New Zealand, confirming its first reported case of HPAI in chickens, also caused by an H7 subtype [20]. Given these recent developments, we examined the epidemiological links of the H7 subtype responsible for recent outbreaks in livestock in Australia and New Zealand to assess the viral evolution and to gain a clearer understanding of the evolving threat in the region.

Sequence retrieval from the GISAID and NCBI databases confirmed that H7 AIV sequences are absent from other Oceanian countries except Australia and New Zealand. This gap likely reflects the limited surveillance infrastructure, sparse poultry populations, and geographical isolation of many Pacific Island nations, which often lack active avian disease monitoring or access to advanced diagnostic techniques such as molecular sequencing [27]. The phylogenetic analysis of HA gene sequences from Australia and New Zealand (Figure 1) revealed clear geographic structuring. Australian AIV H7 sequences formed a distinct and well-supported clade, containing viruses isolated from both poultry and wild birds. New Zealand sequences, by contrast, formed two separate sub-clades with one representing LPAI H7N7 viruses from wild mallards and another comprising HPAI H7N6 viruses from chicken samples collected in 2015 and 2024, respectively. The absence of shared clustering between Australian and New Zealand sequences suggests evolutionary divergence and independent AIV lineages circulating within each country, likely driven by separate introduction events followed by localised evolution. This observation aligns with previous findings by Wille [28], who reported that Australia’s AIV gene pool demonstrates limited gene flow from outside regions and that local wild birds likely serve as reservoirs for persistent endemic viral strains. Similarly, Stanislawek [21] characterised New Zealand’s AIV H7 strains as genetically distinct, suggesting evolutionary isolation and minimal connection to Australian lineages. Geographic and ecological factors likely reinforce this genetic separation. The Tasman Sea functions as a significant biogeographic barrier between Australia and New Zealand, limiting cross-regional transmission. Unlike the Northern Hemisphere, where overlapping migratory flyways facilitate intercontinental spread, Oceania’s avian ecology is characterised by resident and nomadic bird species with limited regional movements [28,29]. As a result, the intermixing of AIV lineages between Oceania and Asia is limited.

Phylogenetic analysis incorporating sequences from Southeast and East Asia (Figure 2) supports this, with AIV H7 sequences from Australia and New Zealand forming independent clades that are genetically distinct from the more diverse Asian lineages. The absence of phylogenetic clustering between Oceanian and Asian H7 sequences suggests limited recent gene flow between regions, particularly in the HA gene segment. This pattern can be attributed to both ecological isolations, stemming from the absence of intercontinental migratory routes, and strict national biosecurity policies. Australia and New Zealand maintain stringent import biosecurity controls and disease monitoring systems, which significantly reduce the risk of viral introduction through the poultry trade or contaminated materials.

Further analysis involving the NA gene provided additional insights. BLAST comparisons indicated that the NA genes of HPAI H7N6 (New Zealand), H7N9, and H7N3 (Australia) shared high sequence similarity with older local LPAI viruses from wild birds, suggesting in situ evolution from endemic gene pools. These findings suggest that viruses of local origin are most likely to have caused the 2024–2025 outbreaks in both countries. However, one exception was noted. The NA gene of the HPAI H7N8 strain isolated in New South Wales (2024) and Victoria, Australia (2025), showed the greatest similarity to N8 sequences from non-H7 subtypes, including H3N8, H4N8, and H6N8 viruses isolated from wild birds in East Asia (2019–2021), with nucleotide identities between 97.30% and 98.09%. This indicates a relatively recent common ancestor and suggests that the NA gene may have entered the Australian gene pool through migratory birds, followed by reassortment with a local H7 HA virus. Although rare, such long-distance viral movement via migratory birds has been documented [28], and the divergence of 2–3% between the Australian and East Asian N8 genes indicates the emergence of a distinct sub-lineage.

The majority of related NA sequences identified through BLAST belonged to non-H7 virus subtypes (e.g., H3, H4, H6), further illustrating the semi-independent evolution of HA and NA genes via reassortment events [30]. This reinforces the notion that while the HA gene primarily drives subtype classification and host–pathogen interactions, NA gene diversity may follow different evolutionary trajectories. Taken together, the evidence indicates that recent HPAI H7 outbreaks in Australia and New Zealand were caused by locally evolved viruses. In Australia, HPAI H7 viruses from 2020 (H7N7) and 2024 (H7N3, H7N9) formed a tight clade, suggesting that they derived from a single ancestral lineage rather than multiple independent incursions. This evolutionary trajectory is consistent with prior outbreaks, where H7 viruses have demonstrated the capacity for in situ mutation and persistence [28]. In New Zealand, the emergence of HPAI H7N6 in 2024 marks the country’s first reported HPAI in poultry. Phylogenetic reconstruction suggests that the 2024 viruses arose from or were closely related to LPAI H7N7 viruses detected in wild mallards in 2015. These two groups formed separate but adjacent clades on the phylogenetic tree, suggesting shared ancestry. The nine-year gap between detections may reflect undetected viral circulation in wild or domestic birds, underscoring gaps in surveillance. The apparent progression from LPAI to HPAI is consistent with known evolutionary mechanisms, where mutations in the HA cleavage site can drive increased pathogenicity [31].

Recent developments show that both Australia and New Zealand have successfully declared the eradication of recent HPAI H7 outbreaks. In Australia, the Department of Agriculture, Fisheries and Forestry (DAFF) confirmed the eradication of H7 as of June 2025, following coordinated national response and biosecurity measures [32,33]. This was further supported by outbreak monitoring updates provided by the Australian Government, affirming that Australia remains free from HPAI H7. Similarly, New Zealand declared the eradication of HPAI H7N6 by December 2024, following the successful containment of outbreaks in Otago, with the Ministry for Primary Industries (MPI) confirming that no active cases of HPAI remain [34].

In parallel with these eradication efforts, both countries have experienced a marked shift in poultry production toward free-range systems, particularly within the commercial egg sector. This transition inherently reduces the effectiveness of on-farm biosecurity by increasing opportunities for direct and indirect contact between poultry and wild birds [17,35]. Modelling and field-based studies have consistently demonstrated that free-range poultry systems carry a higher risk of avian influenza incursion than fully enclosed housing due to greater exposure to wild waterfowl, contaminated surface water, and environmental fomites [35]. Notably, the 2024–2025 HPAI H7 outbreaks in both Australia and New Zealand predominantly affected commercial layer farms operating under free-range or barn-based production systems [20,32,33,34], reinforcing the heightened vulnerability of these systems to spillover from wild reservoirs. When considered alongside our phylogenetic evidence that the outbreak viruses were likely derived from locally circulating wild-bird lineages, this shift in farming practice provides a coherent ecological explanation for the repeated emergence of HPAI H7 in commercial poultry. Collectively, these findings highlight how changes in production systems can directly intersect with viral evolutionary dynamics and further underscore the necessity for continuous, integrated genomic surveillance of avian influenza viruses in wild birds to strengthen early-warning capacity and outbreak prevention.

## 5. Conclusions

This study provides important insights into the epidemiological links of recent HPAI H7 outbreaks in Oceania, specifically in Australia and New Zealand, enhancing our understanding of virus spread and informing future surveillance and control efforts. The results of this study demonstrate that HPAI H7 outbreaks are primarily driven by local viral evolution rather than recent novel introductions from other regions. This local mutation dynamic presents a potential threat not only to the avian population but also to other susceptible livestock species, highlighting the importance of early detection and sustained surveillance from a veterinary public health standpoint. The recent shift toward free-range and barn-based commercial egg production in both countries has increased the vulnerability of poultry to HPAI H7 spillover from wild birds, providing an ecological context for the repeated outbreaks observed in 2024–2025. The data support the existence of distinct, geographically structured AIV lineages shaped by ecological isolation, migratory bird patterns, and stringent national biosecurity policies. Collectively, these findings underscore the importance of ongoing genomic surveillance to detect shifts in pathogenicity that could impact animal health and agricultural biosecurity, particularly as changes in production systems intersect with viral evolutionary dynamics.

## Figures and Tables

**Table 1:** Summary of AIV H7 HA gene sequences from Oceania.

Isolate	Subtype	Location	Year	Host
A/Mallard duck/New Zealand/15.880.121/2015	H7N7	New Zealand	2015	Mallard duck
A/Mallard duck/New Zealand/15.880.80/2015	H7N7	New Zealand	2015	Mallard duck
A/Chicken/NZ/W24_2595/2024(H7N6)	H7N6	New Zealand	2024	Chicken
A/Gallus gallus/Otago/W24_2595/2024	H7N6	New Zealand	2024	Chicken
A/wild waterfowl/South Australia/16-3944344-87/2016	H7N2	Australia	2016	Wild waterfowl
A/wild waterfowl/New South Wales/M18-13121-30/2018	H7N1	Australia	2018	Wild waterfowl
A/wild waterfowl/New South Wales/M18-13121-13/2018	H7N1	Australia	2018	Wild waterfowl
A/wild waterfowl/New South Wales/M18-13121-12/2018	H7N1	Australia	2018	Wild waterfowl
A/grey teal/Victoria/19-01749-185/2019	H7N8	Australia	2019	Grey teal
A/chicken/Lethbridge/20-02865-0008/2020	H7N7	Australia	2020	Chicken
A/chicken/Victoria/20-02865-0008/2020	H7N7	Australia	2020	Chicken
A/chicken/Victoria/10-17/2024	H7N9	Australia	2024	Chicken
A/chicken/Victoria/24-01759-3/2024	H7N3	Australia	2024	Chicken
A/chicken/New South Wales/M24-09739-10/2024	H7N8	Australia	2024	Chicken
A/chicken/Victoria/25-00440-0002/2025	H7N8	Australia	2025	Chicken

**Table 2:** Distribution of AIV H7 sequences from Southeast and East Asia.

Region	Country	Sub-Total	Total
South East Asia	Cambodia	10	
	Vietnam	2	12
	China	865	
	Hong Kong	3	
	Japan	19	
East Asia	North Korea	1	975
	South Korea	42	
	Mongolia	9	
	Taiwan	36	
	Total		987

**Table 3:** HPAI H7Nx virus from recent outbreaks in Australia and New Zealand (2024–2025).

Isolate	Subtype	Location	Year	Host
A/Chicken/NZ/W24_2595/2024(H7N6)	H7N6	New Zealand	2024	Chicken
A/Gallus gallus/Otago/W24_2595/2024	H7N6	New Zealand	2024	Chicken
A/chicken/Victoria/10-17/2024	H7N9	Australia	2024	Chicken
A/chicken/Victoria/24-01759-3/2024	H7N3	Australia	2024	Chicken
A/chicken/New South Wales/M24-09739-10/2024	H7N8	Australia	2024	Chicken
A/chicken/Victoria/25-00440-0002/2025	H7N8	Australia	2025	Chicken

## Data Availability

The data presented in this study are available in the Global Initiative on Sharing All Influenza Data (GISAID) database [https://gisaid.org]. This data were derived from the following resources available in the public domain: the GISAID EpiFlu™ Database [https://platform.epicov.org/] accessed on 10 June 2025.

## Supplementary Materials

The following supporting information can be downloaded at: https://www.mdpi.com/article/10.3390/vetsci12121208/s1, Supplementary Table S1. Avian Influenza Virus H7 sequence metadata from Oceania; Supplementary Table S2. Avian Influenza Virus H7 sequence metadata from East Asia; Supplementary Table S3. Avian Influenza Virus H7 sequence metadata from South East Asia.

## Abbreviations

The following abbreviations are used in this manuscript:

AI	Avian Influenza
AIV	Avian Influenza Virus
HPAI	Highly Pathogenic Avian Influenza
LPAI	Low Pathogenic Avian Influenza
NA	Neuraminidase
HA	Hemagglutinin
H7	Hemagglutinin 7
